# Histone deacetylase 6 and cytoplasmic linker protein 170 function together to regulate the motility of pancreatic cancer cells

**DOI:** 10.1007/s13238-013-0010-3

**Published:** 2014-01-29

**Authors:** Dengwen Li, Xiaodong Sun, Linlin Zhang, Bing Yan, Songbo Xie, Ruming Liu, Min Liu, Jun Zhou

**Affiliations:** 1 Department of Genetics and Cell Biology, College of Life Sciences, Nankai University, Tianjin, 300071 China; 2 Department of Biochemistry, Basic Medical College, Tianjin Medical University, Tianjin, 300070 China

**Keywords:** pancreatic cancer, cell motility, cell migration, cell proliferation, cell cycle

## Abstract

Pancreatic cancer is a devastating disease with the worst prognosis among all the major human malignancies. The propensity to rapidly metastasize contributes significantly to the highly aggressive feature of pancreatic cancer. The molecular mechanisms underlying this remain elusive, and proteins involved in the control of pancreatic cancer cell motility are not fully characterized. In this study, we find that histone deacetylase 6 (HDAC6), a member of the class II HDAC family, is highly expressed at both protein and mRNA levels in human pancreatic cancer tissues. HDAC6 does not obviously affect pancreatic cancer cell proliferation or cell cycle progression. Instead, it significantly promotes the motility of pancreatic cancer cells. Further studies reveal that HDAC6 interacts with cytoplasmic linker protein 170 (CLIP-170) and that these two proteins function together to stimulate the migration of pancreatic cancer cells. These findings provide mechanistic insight into the progression of pancreatic cancer and suggest HDAC6 as a potential target for the management of this malignancy.

## Introduction

Pancreatic cancer is the second leading cause of lethality in the cancer-associated cases of the digestive system. Rapid metastasis to lymph nodes and distant organs is a devastating nature of pancreatic cancer, but the molecular events underlying this remain mysterious (Hezel et al., 2006; Ghaneh et al., 2008). A central process in cancer metastasis is cell motility, which involves drastic cell shape changes driven by cytoskeletal remodeling (Waterman-Storer and Salmon, 1999; Olson and Sahai, 2009; Wells et al., 2013). Both microtubules and actin filaments have been demonstrated to play important roles in cell motility, and their dynamic rearrangement during cell motility is regulated by diverse cytoskeleton-associated proteins (Waterman-Storer and Salmon, 1999; Olson and Sahai, 2009). Cytoplasmic linker protein 170 (CLIP-170) is a microtubule-binding protein that regulates cell motility by modulating microtubule dynamics (Maekawa and Schiebel, 2004). CLIP-170 can bind newly formed plus ends of growing microtubules and rapidly dissociate from the old microtubule lattice (Dragestein et al., 2008). Recently, CLIP-170 has been reported to stimulate angiogenesis and mediate paclitaxel sensitivity in breast cancer (Sun et al., 2012, 2013a). It is unknown whether CLIP-170 is involved in the pathogenesis of pancreatic cancer.

Histone deacetylase 6 (HDAC6) is a member of the class II HDAC family and has two functional deacetylase domains (Valenzuela-Fernandez et al., 2008). Unlike the other HDACs, HDAC6 is localized mainly in the cytoplasm and regulates microtubule dynamics through deacetylating one of the microtubule subunits, α-tubulin (Hubbert et al., 2002; Matsuyama et al., 2002; Zhang et al., 2003). HDAC6 has been shown to participate in a wide range of cellular processes primarily through its deacetylation of α-tubulin, the actin-binding protein cortactin, and the molecular chaperone heat shock protein 90 (Hubbert et al., 2002; Matsuyama et al., 2002; Zhang et al., 2003; Kovacs et al., 2005; Zhang et al., 2007). In addition, HDAC6 can form various complexes with partner proteins to regulate physiological or pathological processes (Parmigiani et al., 2008; Huo et al., 2011; Li et al., 2011). Over the past decade, there has been tremendous effort to develop effective and specific HDAC6 inhibitors for the treatment of human diseases including cancer, although their mechanisms of action remain largely elusive (Aldana-Masangkay and Sakamoto, 2011; Dallavalle et al., 2012). In this study, our data demonstrate that HDAC6 is highly expressed in pancreatic cancer and functions together with CLIP-170 to promote the motility of pancreatic cancer cells, suggesting HDAC6 as a potential target for treating this notorious disease.

## Results

### HDAC6 is highly expressed in human pancreatic cancer

**Figure 1:**
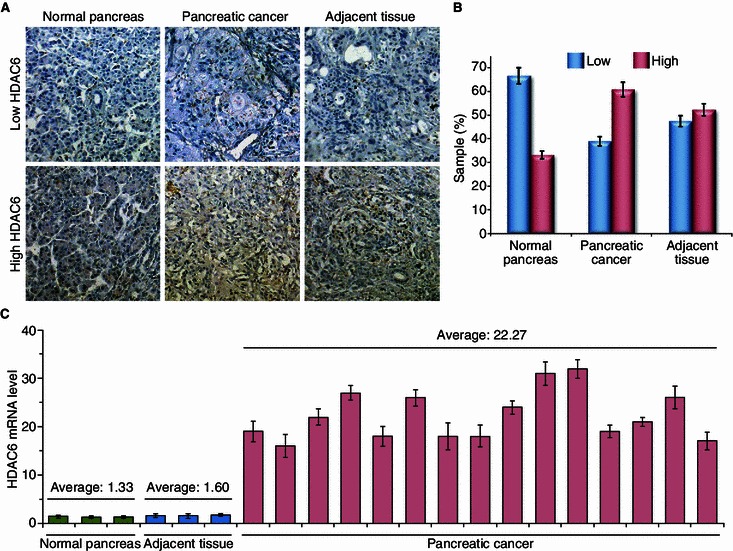
HDAC6 is highly expressed in human pancreatic cancer. (A) Immunohistochemistry of HDAC6 expression in normal pancreas, pancreatic cancer, and adjacent tissue samples. Representative images of samples with low and high expression of HDAC6 are shown. (B) Quantification of normal pancreas, pancreatic cancer, and adjacent tissue samples with low or high expression of HDAC6. (C) Quantitative real-time RT-PCR analysis of HDAC6 mRNA levels in normal pancreas, pancreatic cancer, and adjacent tissue samples

To investigate the potential role of HDAC6 in the pathogenesis of pancreatic cancer, we examined its expression by immunohistochemistry in normal pancreas, pancreatic cancer, and adjacent tissue samples (Fig. 1A). The majority of normal pancreas samples and nearly half of the adjacent tissue samples showed low HDAC6 expression (Fig. 1B). In contrast, a significant increase in HDAC6 expression was observed in pancreatic cancer samples; 38.1% of the cancer samples showed low expression and 61.9% had high expression (Fig. 1B). We then analyzed HDAC6 mRNA levels by quantitative real-time RT-PCR. We found that HDAC6 mRNA expression was up-regulated in 15 out of 15 samples of pancreatic cancer tissues relative to normal pancreas or adjacent tissues (Fig. 1C). The level of HDAC6 mRNA in pancreatic cancer tissues was 16.74-fold and 13.92-fold higher, respectively, than in normal pancreas and adjacent tissues (Fig. 1C).

### Knockdown of HDAC6 expression does not significantly affect pancreatic cancer cell proliferation or cell cycle progression

**Figure 2:**
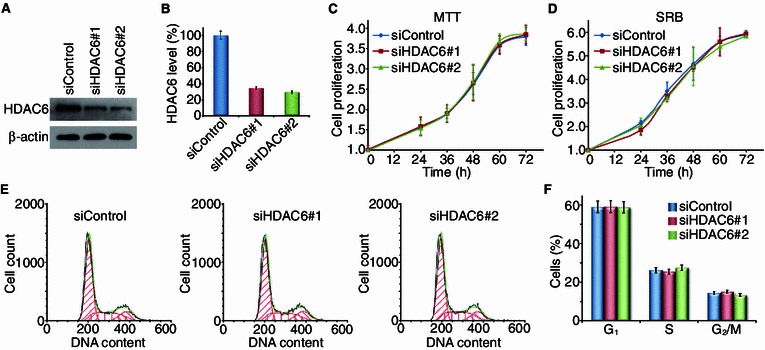
Knockdown of HDAC6 expression does not significantly affect pancreatic cancer cell proliferation or cell cycle progression. (A) Immunoblot analysis of HDAC6 and β-actin expression in PANC-1 cells transfected with control or two different HDAC6 siRNAs for 48 h. (B) Experiments were performed as in (A), and HDAC6 expression levels were quantified and normalized to the control group. (C) Cells were transfected with control or HDAC6 siRNAs, and cell proliferation was examined with the MTT assay. (D) Cells were transfected with control or HDAC6 siRNAs and cell proliferation was examined by sulforhodamine B staining. (E) Cells were transfected with control or HDAC6 siRNAs and cell cycle progression was examined by flow cytometry. (F) Experiments were performed as in (E), and the percentage of cells in G_1_, S, and G_2_/M phases were analyzed

Given the high expression of HDAC6 in pancreatic cancer tissues, we speculated that it might promote the proliferation of pancreatic cancer cells. To test this, we inhibited HDAC6 expression in pancreatic cancer cells by using two different siRNAs, #1 targeting the coding region and #2 targeting the untranslated region. Immunoblot analysis showed that both of the two siRNAs worked effectively (Fig. 2A and 2B). Cell proliferation was then examined by sulforhadamine B staining assay, which is widely used for cell density determination based on the measurement of cellular protein content (Vichai and Kirtikara, 2006). We found that siRNA-mediated knockdown of HDAC6 expression did not obviously affect pancreatic cancer cell proliferation (Fig. 2D). Similar results were achieved by using MTT staining assay (Fig. 2C), which is based on the reduction of yellow tetrazole to purple formazan in living cells (Berridge et al., 2005). We also investigated the effects of HDAC6 siRNAs on the cell cycle progression of cancer cells by flow cytometric analysis of DNA content. As shown in Fig. 2E and 2F, knockdown of HDAC6 expression did not have obvious effects on the distribution of cells in G_1_, S, and G_2_/M phases. Together, these results demonstrate that knockdown of HDAC6 expression does not affect the proliferation or cell cycle progression of pancreatic cancer cells.

### Decrease of HDAC6 expression or inhibition of its activity impairs the motility of pancreatic cancer cells

**Figure 3:**
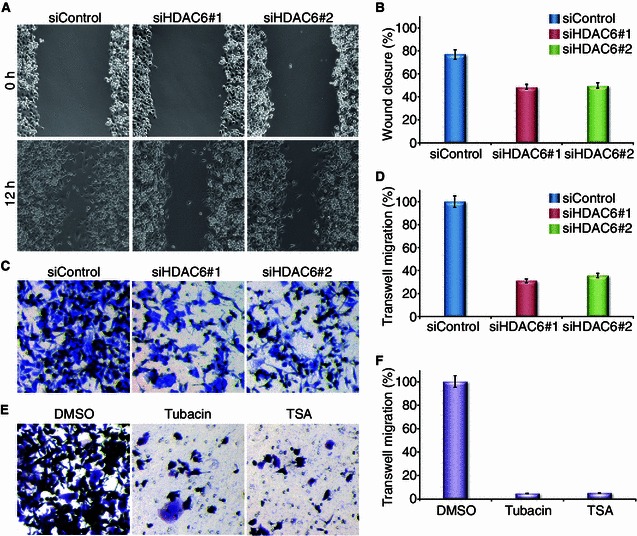
Decrease of HDAC6 expression or inhibition of its activity impairs the motility of pancreatic cancer cells. (A) PANC-1 cells transfected with control or HDAC6 siRNAs were scratched, and wound margins were imaged 0 h and 12 h later. (B) Experiments were performed as in (A), and the extent of wound closure at 12-h point was quantified by measuring the wound area compared with the initial wound area. (C) Cells transfected with control or HDAC6 siRNAs were plated onto the inside of the transwell insert, and the insert was placed in a 24-well plate containing conditional media. After 18 h, cells migrated to the underside of the insert were stained with crystal violet. (D) Experiments were performed as in (C), and the amount of migrated cells was measured and normalized to the control group. (E) Transwell migration experiments were performed as in (C), except that cells were treated with tubacin or TSA. (F) Experiments were performed as in (E), and the amount of migrated cells was measured and normalized to the control group

To gain more insight into the potential functions of HDAC6 in pancreatic cancer, we examined whether it is involved in the motility of pancreatic cancer cells. By scratch wound assays, we found that HDAC6 siRNAs remarkably compromised the ability of pancreatic cancer cells to migrate into the wound area (Fig. 3A and 3B). Transwell migration assays further revealed that HDAC6 siRNAs dramatically reduced the amount of cancer cells to migrate across the porous membrane (Fig. 3C and 3D). To investigate whether the role of HDAC6 in mediating pancreatic cancer cell migration requires its catalytic activity, we treated cells with tubacin, a potent and selective HDAC6 inhibitor (Haggarty et al., 2003), and trichostatin A (TSA), a pan-HDAC inhibitor (Lindemann et al., 2004). As shown in Fig. 3E and 3F, both tubacin and TSA effectively suppressed the transwell migration ability of pancreatic cancer cells.

### HDAC6 overexpression enhances pancreatic cancer cell motility without affecting cell proliferation

**Figure 4:**
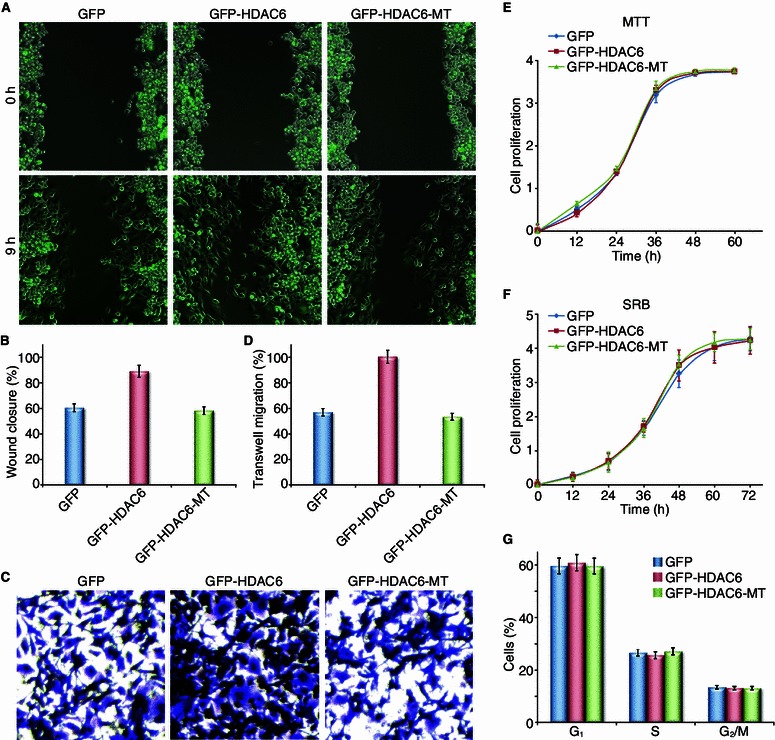
HDAC6 overexpression enhances pancreatic cancer cell motility without affecting cell proliferation. (A) PANC-1 cells transfected with GFP, GFP-HDAC6, or GFP-HDAC6-MT were scratched, and wound margins were imaged 0 h and 9 h later. MT, catalytically inactive mutant. (B) Experiments were performed as in (A), and the extent of wound closure was quantified by measuring the wound area compared with the initial wound area. (C) Cells transfected with the indicated plasmids were plated onto the inside of the transwell insert, and the insert was placed in a 24-well plate containing conditional media. After 18 h, cells migrated to the underside of the insert were stained with crystal violet. (D) Experiments were performed as in (C), and the amount of migrated cells was measured. (E) PANC-1 cells were transfected with the indicated plasmids, and cell proliferation was examined with the MTT assay. (F) Cells were transfected with the indicated plasmids, and cell proliferation was examined by sulforhodamine B staining. (G) Cells were transfected with the indicated plasmids, and cell cycle progression was examined by flow cytometry

To corroborate the role of HDAC6 in the motility of pancreatic cancer cells, we studied the effects of its overexpression. Strikingly, both scratch wound assay and transwell migration assay showed that overexpression of HDAC6 stimulated pancreatic cancer cell migration (Fig. 4A–D). This effect of HDAC6 was abrogated by overexpression of a catalytically inactive mutant of HDAC6, which harbors H216A and H611A mutations in the deacetylase domains (Fig. 4A–D). Collectively, these data reveal that the deacetylase activity of HDAC6 is important for its role in promoting pancreatic cancer cell migration. We also performed MTT and SRB assays and flow cytometry to analyze the effects of HDAC6 overexpression on pancreatic cancer cell proliferation and cell cycle progression. As shown in Fig. 4E–G, in agreement with the HDAC6 siRNA results, overexpression of HDAC6 or its catalytically inactive mutant did not significantly affect the proliferation or cell cycle progression of pancreatic cancer cells.

### HDAC6 interacts with CLIP-170

**Figure 5:**
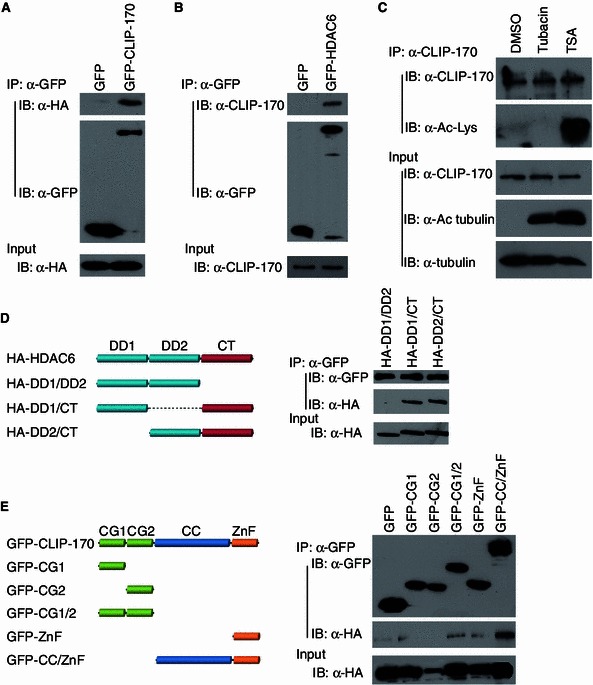
HDAC6 interacts with CLIP-170. (A) Cells were transfected with HA-HDAC6 and GFP or GFP-CLIP-170. Immunoprecipitation (IP) was then performed to examine the interaction between HA-HDAC6 and GFP-CLIP-170. (B) Cells were transfected with GFP or GFP-HDAC6, and immunoprecipitation was performed to examine the interaction between GFP-HDAC6 and endogenous CLIP-170. (C) Cells were treated with DMSO, tubacin, or TSA. Immunoprecipitation and immunoblotting were then performed with antibodies against CLIP-170 and acetylated lysine (Ac-Lys). Cell lysates were immunoblotted with antibodies against α-tubulin, acetylated α-tubulin (Ac-tubulin), and CLIP-170. (D) Cells were transfected with GFP-CLIP-170 and various forms of HDAC6 tagged with HA. Cell lysates and anti-GFP immunoprecipitates were immunoblotted with anti-GFP and anti-HA antibodies. Left panel shows various forms of HA-HDAC6 used. DD, deacetylase domain; CT, carboxyl terminus. (E) Cells were transfected with HA-HDAC6 and various forms of CLIP-170 tagged with GFP. Cell lysates and anti-GFP immunoprecipitates were immunoblotted with anti-GFP and anti-HA antibodies. Left panel shows various forms of CLIP-170 used. CG, CAP-Gly; CC, coiled coil; ZnF, zinc finger

We have previously found that HDAC6 associates with microtubule end-binding proteins in endothelial cells (Li et al., 2011). This finding prompted us to examine the potential interaction of HDAC6 with CLIP-170, a protein localized primarily at the ends of growing microtubules. Immunoprecipitation assays revealed that HDAC6 interacts with both exogenous and endogenous CLIP-170 in cells (Fig. 5A and 5B). Treatment with TSA, but not tubacin, could increase CLIP-170 acetylation, although both tubacin and TSA greatly enhanced the level of α-tubulin acetylation (Fig. 5C), indicating that CLIP-170 is not a substrate of HDAC6. To identify the domain(s) on HDAC6 that mediates its interaction with CLIP-170, plasmids that express various truncated forms of HDAC6 were transfected together with GFP-CLIP-170. By immunoprecipitation we found that the carboxyl terminus of HDAC6 was required for its interaction with CLIP-170, as the truncated form of HDAC6 lacking the carboxyl terminus was unable to bind CLIP-170, while the mutants containing this region were able to bind (Fig. 5D). Similarly, by using truncated forms of CLIP-170, we found that both the coiled coil domain and the zinc finger domain of CLIP-170 were necessary for its interaction with HDAC6 (Fig. 5E).

### HDAC6 coordinates with CLIP-170 to regulate pancreatic cancer cell migration

**Figure 6:**
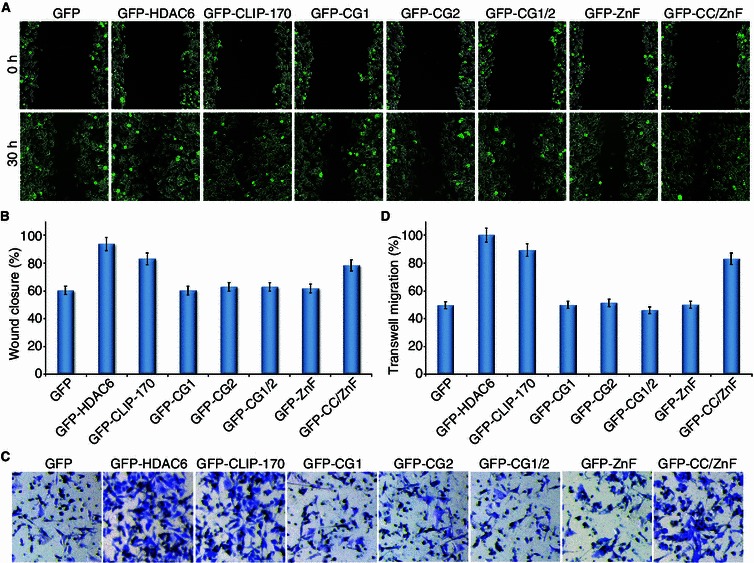
HDAC6 coordinates with CLIP-170 to regulate pancreatic cancer cell migration. (A) PANC-1 cells transfected with HDAC6 siRNA and GFP, GFP-HDAC6, or GFP-CLIP-170 (full-length or various truncated forms) for 48 h were scratched, and wound margins were imaged 0 h and 30 h later. (B) Experiments were performed as in (A), and the extent of wound closure was quantified. (C) Cells were transfected as in (A), and transwell migration experiments were performed and cells migrated to the underside of the insert were stained with crystal violet. (D) Experiments were performed as in (C), and the amount of migrated cells was quantified

We then investigated whether the interaction with CLIP-170 is involved in the function of HDAC6 in pancreatic cancer cell migration. To test this, pancreatic cancer cells were transfected with HDAC6 siRNA and GFP, GFP-HDAC6, or GFP-CLIP-170. By scratch wound assays, we found that both GFP-HDAC6 and GFP-CLIP-170 could efficiently rescue HDAC6 siRNA-induced cell migration defects (Fig. 6A and 6B). In addition, the CLIP-170 mutant containing the HDAC6-interacting domain could partially rescue the migration defects, but the mutants lacking the HDAC6-interacting domain were unable to rescue (Fig. 6A and 6B). Similar results were obtained by transwell migration experiments (Fig. 6C and 6D). Taken together, these results suggest that HDAC6 acts in concert with CLIP-170 to promote the motility of pancreatic cancer cells.

## Discussion

Cell motility is a process critical for the metastasis of pancreatic cancer as well as the other cancer types (Waterman-Storer and Salmon, 1999; Olson and Sahai, 2009; Wells et al., 2013). Understanding the regulation of cell motility therefore might expedite the development of novel therapeutic approaches. Most migrating cells are polarized, due to directed membrane trafficking and asymmetrical redistribution of the cytoskeleton and signaling molecules (Etienne-Manneville, 2008). While the protrusive activity of migrating cells largely depends on actin polymerization, the establishment and maintenance of cell polarity require coordinated actions of actin filaments and microtubules (Rodriguez et al., 2003). Microtubule dynamics are regulated exquisitely in cells by a repertoire of microtubule-binding proteins, including proteins that regulate the assembly and organization of microtubules and motor proteins that mediate the transport of organelles and vesicles (Amos and Schlieper, 2005). In this study, we demonstrate that the microtubule-associated deacetylase HDAC6 interacts with the microtubule end binding protein CLIP-170 to stimulate pancreatic cancer cell motility. This finding suggests that cell motility and many other microtubule-mediated cellular processes may depend on the coordination of various microtubule-binding proteins.

As a cytoplasmic HDAC, HDAC6 has been implicated in the regulation of many cancer-associated cellular events and signaling pathways, making it an attractive target for cancer chemotherapy (Aldana-Masangkay and Sakamoto, 2011; Dallavalle et al., 2012). The present study reveals that pancreatic cancer samples have higher HDAC6 expression, at both protein and mRNA levels than normal pancreas and adjacent tissues. At present, the mechanisms of how HDAC6 expression is elevated in pancreatic cancer cells are unclear and warrant further investigation. Our study also reveals that HDAC6 is involved in the motility but not the proliferation or cell cycle progression of pancreatic cancer cells, which is in agreement with previous findings in other cell types (Haggarty et al., 2003). Together, these results suggest that the role of HDAC6 in pancreatic cancer may mainly lie in its metastasis instead of tumor growth. Our findings indicate that HDAC6 might have value in the diagnosis of pancreatic cancer. In addition, given that HDAC6 inhibitors are undergoing clinical studies for certain diseases, the data shown in this study suggest an importance for investigating the effectiveness of HDAC6 inhibitors for pancreatic cancer treatment.

The localization and functions of CLIP-170 are known to undergo regulation by posttranslational modifications such as phosphorylation (Yang et al., 2009; Li et al., 2010; Nakano et al., 2010; Takashima, 2011). Our study shows that CLIP-170 can be acetylated in cells; however, the level of CLIP-170 acetylation is not affected by the HDAC6 inhibitor tubacin, although the deacetylase activity is necessary for the role of HDAC6 in regulating pancreatic cancer cell motility. Given that CLIP-170 could largely rescue HDAC6 siRNA-induced cell migration defects, it is tempting to speculate that CLIP-170 may play a scaffolding role to facilitate HDAC6 actions toward cell motility. It is also worthy of note that in addition to tracking the plus ends of growing microtubules, CLIP-170 has been shown to mediate the interaction between endocytic vesicles and microtubules (Pierre et al., 1992). It is possible that HDAC6 and CLIP-170 may function together at the interface between endocytic vesicles and microtubules to facilitate cell polarization and motility.

## Materials and methods

### Materials

Sulforhodamine B and 3-(4,5-dimethyl-2-thiazolyl)-2,5-diphenyl-2H-tetrazolium bromide (MTT) were purchased from Sigma-Aldrich. Antibodies against CLIP-170 and HDAC6 (Santa Cruz Biotechnology), β-actin and HA (Sigma-Aldrich) and GFP (Roche) were obtained from the indicated sources. Horseradish peroxidase-conjugated secondary antibodies were from Amersham Biosciences. GFP-HDAC6, HA-HDAC6, and GFP-CLIP-170 expression plasmids and various truncated forms were constructed by PCR using pEGFPN1 and pCMV-HA vectors as described previously (Zhou et al., 2009; Li et al., 2011; Sun et al., 2012, 2013a). HDAC6 and luciferase control siRNAs were synthesized by Invitrogen.

### Cell culture and transfection

PANC-1 human pancreatic cancer cells were obtained from the American Type Culture Collection and cultured in DMEM medium supplied with 10% fetal bovine serum at 37°C in a humidified atmosphere with 5% CO_2_. Plasmids were transfected to cells by using the polyethyleneimine reagent (Sigma-Aldrich), and siRNAs were transfected with the Lipofectamine 2000 reagent (Invitrogen).

### Immunoblot analysis

Cells were lysed in a buffer containing 1% Triton X-100, 150 mmol/L NaCl, and 50 mmol/L Tris (pH 7.5). Proteins were resolved by sodium dodecyl sulfate-polyacrylamide gel electrophoresis and transferred onto polyvinylidene difluoride membranes (Millipore). The membranes were blocked in Tris-buffered saline containing 0.2% Tween 20 and 5% fat-free dry milk and incubated with primary antibodies and horseradish peroxidase-conjugated secondary antibodies, respectively. Specific proteins were visualized with enhanced chemiluminescence detection reagent according to the manufacturer’s instructions (Pierce Biotechnology).

### Fluorescence microscopy

Cells grown on glass coverslips were fixed with 4% paraformaldehyde for 30 min at room temperature. Cells were blocked with 2% bovine serum albumin in phosphate-buffered saline (PBS), and coverslips were mounted with 90% glycerol in PBS and examined with an Axio Observer A1 fluorescence microscope (Carl Zeiss).

### Immunohistochemistry

Human pancreatic tissues were obtained from patients undergoing surgical resection at Shanxian Dongda Hospital. Paraffin-embedded tissue sections were cut, deparaffinized, and rehydrated with xylene and graded alcohols. Antigen retrieval was performed in 5 mmol/L citrate buffer. After inactivation of endogenous peroxidase with 3% H_2_O_2_, the sections were blocked with goat serum and incubated with primary antibody. The sections were then incubated with biotinylated secondary antibody and streptavidin-biotin-peroxidase, and diaminobenzidine was used as a chromogen substrate. The sections were counterstained with hematoxylin. Protein expression was graded based on the intensity of staining and the percentage of stained cells as described previously (Sun et al., 2013b).

### Quantitative real-time RT-PCR

Total RNA was isolated using the TRIzol reagent (Invitrogen), and quantitative real-time RT-PCR was performed using the SYBR Premix Ex Taq reagent (Takara) according to the manufacturer’s instructions.

### Flow cytometry

Cells were centrifuged, washed twice with ice-cold PBS, and fixed in 70% ethanol. Tubes containing the cell pellets were stored at -20°C for at least 24 h. After this, the cells were centrifuged at 1000 *g* for 10 min, and the supernatant was discarded. The pellets were resuspended in phosphate/citrate buffer (pH 7.5) at room temperature for 30 min. Cells were then washed with PBS and incubated with propidium iodide (20 μg/mL)/RNaseA (20 μg/mL) in PBS for 30 min. Samples were analyzed on a Coulter Elite flow cytometer (Beckman Coulter).

### Cell motility assays

To analyze cell migration by wound healing, confluent monolayers of cells cultured in 24-well plates in serum-free medium were scratched with a 10-mL pipette tip to generate the wound. Cells were washed with PBS to remove the debris. Phase-contrast photographs of the wound were taken at different time points to determine the extent of wound closure. Transwell migration assays were performed as described previously (Shi et al., 2012). Briefly, cells suspended in serum-free medium were added to the inside of the transwell insert precoated with matrigel, and the insert was then placed in a 24-well plate containing conditioned media. After 18 h, cells on the inside of the transwell insert were removed with a cotton swab, and cells on the underside of the insert were fixed with 4% paraformaldehyde and stained with crystal violet solution.

### Cell proliferation assays

For sulforhodamine B staining, cells were seeded at 1 × 10^4^ cells per well in 96-well tissue culture plates. Cells were fixed with 10% trichloroacetic acid for 1 h in 4°C and stained with 0.4% sulforhodamine B dissolved in 1% acetic acid at different time points. The cells were then washed with 1% acetic acid to remove unbound dye. The protein-bound dye was extracted with 10 mmol/L Tris base to determine the optical density at 490 nm wavelength. For MTT staining, 1 × 10^4^ cells were plated in each well of 96-well tissue culture plates. MTT reagent in PBS was added to each well at different time points, and the cultures were incubated for an additional 4 h. DMSO was added after the MTT solution was removed. The optical density was then determined at 562 nm wavelength.

## Abbreviations

CLIP-170: cytoplasmic linker protein 170
HDAC6: histone deacetylase 6
MTT: 3-(4,5-dimethyl-2-thiazolyl)-2,5-diphenyl-2H-tetrazolium bromide
PBS: phosphate-buffered saline
TSA: trichostatin A
